# Microchips and sneakers: Bilateral trade, shifting power, and interstate conflict

**DOI:** 10.1177/00223433231153902

**Published:** 2023-04-16

**Authors:** Yuleng Zeng

**Affiliations:** Department of Political Science, University of Salzburg

**Keywords:** economic interdependence, network analysis, strategic goods, trade externality

## Abstract

Strong commercial ties promote peace as states shun the opportunity costs of economic disruption. However, trade also enriches and empowers states, rendering them more capable of enforcing long-term settlements. Given economic disruption does not last forever, countries can be incentivized to trade short-term economic losses for long-term political or territorial gains. This trade-off can restrict or even reverse the pacifying effect of commerce as it renders states incapable of committing to existing peaceful deals. I argue the scope condition hinges on the existing power imbalance and the security externalities of trade, defined as states’ abilities to translate trade gains into (potential) military power. For countries where the existing power gap is not extreme, the impact of bilateral strategic trade is contingent upon a country’s trade externality relative to its opponent’s. Although increased bilateral trade can be peace-promoting when the relative externality is small, the pacifying effects can dissipate as a relatively weaker state becomes more capable of exploiting trade gains. Building on recent work in network analysis, I propose a new measurement of trade externalities to test the above theory and find supporting results.

Strong commercial ties can help promote peace as they bind nations in a mutually dependent relationship. As such, states are less likely to fight and risk the opportunity costs of economic disruption (Gartzke, Li & Boehmer, 2001; Polachek & Xiang, 2010). But history is also replete with cases where increasing bilateral trade can stoke (as well as reduce) conflict,^1^ the relationship between the USA and China being a prominent recent example. Commercial interests and the related restraining effects were clearly behind the US policy toward China in the 1990s. Yet in recent years we witness more tensions simmering on both sides. Many pundits and strategists lament trade can no longer anchor the relationship between the United States and China and fear that the level of threats of limited military conflict between them has become worryingly high (Economist, 2019; Bo, 2020). If trade promotes peace by increasing opportunity costs, then the profits of peace and potential economic losses of conflict are clearly much higher nowadays. The two countries’ trade volume in 1990 was 3.6 times larger than in 1980. By contrast, their bilateral trade has expanded over 100-fold from 1980 to 2010.^2^ With such a dramatic increase in bilateral trade, if the rationale of peace via trade holds we should generally expect a lower likelihood of costly conflict (i.e. conflict involving the use of force).

It is puzzling why the earlier relatively small increase in bilateral trade restrained, while the later massive amount of increase cannot restrain. In essence, this puzzle concerns the scope conditions of bilateral trade’s pacifying effects, which are critically important for the theoretical development of commercial liberalism. It is also of vital importance to global peace because the results can only be disastrous if two great powers such as the USA and China cannot manage their rivalry despite extraordinarily strong commercial interests. One key reason for the recent conflict is that concerns about security ‘mattered less when China exported tennis shoes and televisions rather than microchips’ (Economist, 2019). Granted, there are other issues such as China’s unfair trade practices. But these have been tolerated over the past few decades (Clarke & Johnston, 1999); focusing too much on these specifics would obscure the underlying changes that render previous policies unsustainable. Projects such as Made in China 2025 are concerning for the United States because China’s improvement in manufacturing capacity and technology innovation can be transferred into expanded military power (Fravel, 2019). Indeed, some scholars even advocate that it is high time that the USA should work with its allies to control dual-use exports to China and prepare for more bounded and competitive international orders (Mearsheimer, 2019). At the core of this growing tension from tennis shoes to microchips is the connection between trade, power, and security.

This tension between the USA and China is not *sui generis* as it can also apply to other cases of adversaries. Without an in-depth analysis of the underlying mechanism, we are missing a crucial lens to fully understand the tensions between countries that trade heavily with each other^3^ and may even be misguided when crafting policies to effectively manage them. To better address the puzzle, I argue more attention should be given to the *security externalities* of trade as well as the resulting power shifts over time. I define the security externalities of trade as states’ increased abilities to translate trade gains into (potential) military power due to the expansion of trade. Building on this conceptualization, I contend there is a scope condition under which increased trade could enrich and empower states at different paces, exacerbate security concerns, and ultimately increase the likelihood of costly conflict.

My main argument is that the pacifying effects of increased bilateral strategic trade^4^ will dissipate or even be reversed when a weaker country’s trade externality relative to an opponent’s is large enough and the existing power gap between the two is not extreme. When the existing power imbalance is large, the power shifts introduced by trade externality are unlikely to cause security concerns. As such, the pacifying effects of bilateral trade hold regardless of relative externality. However, when the existing power gap is small, then states can be concerned by potential power shifts. In this scenario, when a weaker state becomes relatively more capable of exploiting trade gains, the security concerns will loom larger. In the meantime, the options of using economic coercion to slow down or reverse power shifts will be less effective or even counterproductive. As a result, the likelihood of costly conflict will increase as states trade more strategic goods.

This article makes a number of contributions. First, it advances the theoretical development of commercial liberalism. The two dominant theories in the field, opportunity costs and costly signaling, are modeled without accounting for the impact of time. My theory, by contrast, specifically examines how trade relations and states’ power shifts over time affect their strategic interactions. It also advances the theory linking uneven trade gains to security dilemmas and identifies a more stringent scope condition: uneven gains lead to heightened tensions only when the power shifting impact is large enough and when the existing power gap is moderate. In other words, it is not the unevenness per se that makes conflict more likely, but rather how relative externality interacts with the existing power balance.

Second, although theoretical arguments about trade externalities are ample, there has been limited work to empirically capture the concept. As a result, empirical work along this line of literature has struggled to gain traction. Building on the recent advancement in trade network studies, this article pushes the concept to further incorporate commodity heterogeneity and states’ integration into the global strategic trade networks, providing a first cut to empirically quantify the security externalities of trade. This measurement can be more widely utilized by researchers broadly interested in international security and political economy.

Finally, it holds important implications for studies of major power competition and strategic rivalries. For example, consider the recent rise of China. With the advance of globalization and China’s economic progress, the relative externality between China and the United States is shifting toward the condition where increasing bilateral trade can stoke costly conflict. This study suggests that instead of stepping up economic containment or building more restrictive international orders, encouraging technology competition and innovation can help propel the relative externality to travel out of the danger zone in a more peaceful manner (Weiss, 2022).

This policy implication is not limited to hegemonic competition as it can also be applied to other potential adversaries, such as Ukraine and Russia, Greece and Turkey, or Thailand and Myanmar, which have also witnessed higher military tensions despite increased trade in recent years. For instance, my theory suggests that the benefits of trade could not prevent Russia’s invasions of Ukraine in 2014 and 2022 due to the combined impact of their relatively small gaps in military power and trade externality. At the time of writing, events are still unfolding on the battlefield, but they have clearly revealed the weakness of the Russian military. The international community can further help Ukraine defend itself by providing both consistent support in military aid and, importantly, also long-term economic assistance in rebuilding and boosting its economy and trade externality.

## When bilateral trade does (not) pacify

Trade–conflict studies can be broadly divided between liberals contending for the pacifying effects of trade and scholars who argue otherwise. Commercial liberalism argues that stronger economic ties can help promote peace because interstate conflict disrupts normal economic exchange, thereby generating ex ante incentives for states to avoid the opportunity costs (Polachek & Xiang, 2010). In addition, stronger economic ties can alleviate the problems of incomplete information, as states that are willing to endure (or risk) higher costs can better signal their resolve (Gartzke, Li & Boehmer, 2001). Unifying these two main theories, Zeng (2020) suggests that in situations of one-shot interaction both the opportunity costs and signaling mechanisms work; since the bargaining environment allows states to inform and coerce simultaneously, the prospect of peace should improve as bilateral trade volume increases.

However, there is also a line of studies that caution against accepting the pacifying effects of bilateral trade wholesale. Some scholars believe that higher economic dependence almost always leads to conflict. In anarchy, states often worry about the economic vulnerability that high interdependence can entail (Barbieri, 1996), and, fearing vital goods being cut off, they may use force to secure their supply (Mearsheimer, 1992). Others argue that depending on the scope conditions, the effects of economic dependence can be either pacific or conflictual (or non-existent). For instance, increased dyadic trade can aggravate conflict when a state’s trade partner faces proportionally lower exit costs (Crescenzi, 2003; Peterson, 2014). States motivated by economic competition and market power goals may also take aggressive actions to achieve their ambitions (Chatagnier & Kavaklı, 2017; Gent & Crescenzi, 2021).^5^

Departing from the focus on existing economic ties, some studies highlight the importance of states’ expectations about future dependence. Copeland (2015) argues if states have positive expectations of future trade, they will strive to keep the benefits of commerce and avoid the opportunity costs of economic disruption. However, if states have negative expectations, namely they will be cut off from trade and investment, then to avoid future economic decline and loss of bargaining power, they will find the use of force today more attractive. Building on this rationale, Monteiro & Debs (2020) show that stronger states may fear weaker states’ economic growth and impose constraints on the latter’s access to markets and resources, resulting in economic hold-up problems. If stronger states cannot credibly commit to grant weaker ones the access, then weaker states may find war a better choice to eliminate the hold-up.

## Trade, power, and security

The above studies can offer some explanations for the security tensions discussed at the beginning of the article. But they all focus on the coercive side of economic dependence and therefore still do not fully address the power competition rationale driven by a weaker state’s improved technology and manufacturing capacity. To capture the underlying tensions and further the trade–conflict studies, we need to strengthen the link between trade, power, and security.

Instead of focusing solely on the costs and coercive effects of trade, some earlier studies have highlighted the possible beneficial aspect. Specifically, free trade can create positive security externalities: as states trade more they become more efficient at utilizing resources, resulting in a stronger economy and military power (Baldwin, 1985; Gowa & Mansfield, 1993). For example, Russia’s ambition to reform and revive its military was only able to be earnestly started in the early 2000s as the economy got a major boost from rising commodity prices (Larson & Shevchenko, 2010). International trade can also help states improve their technological know-how. For instance, commercial satellite launching experience since the 1980s helped China modernize its missile weapons (Meijer, 2016).

These positive externalities of trade often vary across countries and over time given the differences in the composition and networks of trade. Such differences and changes in capacities to utilize trade gains may undermine the pacifying effects of trade because the economic causes of security concerns are inherently tied with power shifts and the (potential) use of force. The ‘relative gains’ argument, for instance, is built on how uneven gains from trade can lead to military (dis)advantages (Grieco, 1990; Gowa, 1994).^6^ The hegemonic stability and power transition theories both highlight the impact of different rates of economic growth (Organski & Kugler, 1980; Gilpin, 1981). Finally, formal theories suggest that if two adversaries’ military power changes at different rates, then peaceful deals today may not be attractive for one or both parties as the security concerns tomorrow loom larger (Powell, 1999; Krainin, 2017; Fearon, 2018).

## Relative externality and the shadow of the future

The previous section suggests in examining the mechanism connecting trade and power shifts we need to incorporate three moving parts together: (a) commerce can enrich and empower states over time; (b) states differ in their abilities to exploit the gains from trade; (c) the prospective shift of military power due to the change of bilateral trade can affect the current likelihood of costly conflict. The research design section offers more discussions about the concept and measurement of (a) and (b). For now, it is sufficient to assume that they hold and I will refer to (a) as ‘trade externality’ (i.e. states’ increased (potential) military power due to the expansion of trade). Throughout the rest of the article, I will also use the term ‘relative externality’ to refer to a weaker state’s trade externality relative to its stronger counterpart.

I argue that when we consider all three parts together the marginal effects of increased bilateral trade are conditioned on relative externality and the existing power imbalance/gap. When the existing power imbalance is too extreme, the impact of power shifts (if there are any) would be too limited and the pacifying impact of trade should not be contingent upon relative externality. However, when the gap of power is not large, security concerns introduced by potential power shifts will loom larger: the pacifying effects of bilateral trade can dissipate or even be reversed as relatively weaker states become more capable of exploiting the gains from trade.

First, when the power gap is extremely large, the potential shifts of power introduced by trade externality would be too small to cause any security concerns. Often, the militarily much weaker states are also less capable of exploiting the gains from trade (see the distribution in the right panel of Figure 2). For instance, countries such as Haiti or Grenada will not pose direct security threats toward the USA by any stretch of imagination. Even in the cases where a weaker state’s trade externality is higher, the potential power shift is often negligible given the extreme power imbalance. For instance, based on my externality measurement the Philippines was more capable than China of exploiting the gains from trade during the Cold War. But it is hard to imagine that this would have created any security concerns for China as their bilateral strategic trade expanded.

Second, when the power imbalance is not extreme, there can be some real concerns that a relatively weaker opponent today may become strong enough tomorrow to pose security threats. This is particularly the case since countries of roughly equal powers are more likely to be concerned about repeated confrontations (Senese & Vasquez, 2008: 12).^7^ In this scenario, if the weaker state is relatively less capable of exploiting the gains from trade, then the power imbalance either remains at the status quo or shifts further to favor the stronger state. If so, increasing bilateral strategic trade can strengthen the pacifying effects of commerce as it augments the existing deterrence and leverage of the stronger side.

However, if the relative externality is large enough, that is, the weaker state can benefit proportionally more from free trade, then the power shift introduced by trade will further close the existing gap. In this scenario, the security concerns would loom larger for the stronger state because they would expect reducing bargaining leverage over time. The relatively weaker state, emboldened by its trajectory of improving military capability, would be more likely and able to resist the policy demands from the stronger counterpart. In this case, the anxious versus emboldened dyad ends up not wanting to strike/maintain a peaceful deal today and instead prefers to draw the lottery of costly conflict.

One potential counter-argument is that the stronger state can choose to restrict strategic trade or apply broader economic coercion to reduce the weaker state’s trade externality. But economic sanctions may increase the risks of conflict given the limits of credibility (Lektzian & Sprecher, 2007) or the heightened tensions of ‘trade–security spiral’ (Copeland, 2015: 45). Even in situations where they do not, economic coercion or containment is less likely to work successfully if the relatively weaker states become more integrated into global trade networks and more capable of exploiting the gains from trade (Peterson, 2020). For instance, the long peace between the United States and the Soviet Union during the Cold War can at least be partially attributed to the former’s ability to contain the USSR and to deny it any meaningful trade externality (Gaddis, 2005; Blackwill & Harris, 2016). In comparison, economic containment toward China these days may not be that productive given its abilities to secure alternative suppliers and markets in strategic goods (Meijer, 2016; Kim, 2019).

Taken together, the above discussions suggest that when the relative externality is small, it is less necessary for the stronger state to use force since it expects the opponent to become relatively weaker over time and it can also apply restrictive economic measures more effectively if needed. When the relative externality is large enough, increasing strategic trade in this case can exacerbate security concerns and stoke costly conflict since it can augment adverse power shifts (for the stronger state) and non-military threats or coercion may not be credible or effective any longer. Therefore, I have the following two hypotheses.

*Hypothesis 1:* When the existing power gap is small, the marginal effects of increased bilateral strategic trade on costly conflict are contingent on relative externality: negative (peace-promoting) when the relative externality is small; and positive (conflict-stoking) when the relative externality is large.
*Hypothesis 2:* When the existing power gap is large, the marginal effects of increased bilateral strategic trade on costly conflict are negative and are not contingent on relative externality.

## Research design

To test the hypotheses, I focus on non-directed politically relevant dyads from 1962 to 2009. In the main models, I separate the data into two samples (small power gap vs. large power gap). Since I focus on the endogenous shift of power, I follow Debs & Monteiro (2014) and use only the endogenous components (military personnel and spending) of the Composite Index of National Capabilities (CINC) index (Singer, Bremer & Stuckey, 1972) to measure states’ power.^8^

Using the endogenous power variable, I first assign state 1 to be the relatively weaker side to establish the non-directed data structure and calculate the power ratio (state 1’s endogenous power divided by the aggregate of state 1’s and state 2’s). Within each year, I assign dyads of the top 25th percentile of the power ratio variable into the small power gap sample and the rest as having a large power gap.^9^ I choose to dichotomize the power ratio variable given my theoretical expectation, the ease of interpretation, and that the existing endogenous power measure is still a rough proxy.^10^

### Costly conflict

To measure the dependent variable, I choose to use the Militarized Interstate Dispute (MID) data (1918–2010). Given the potential issues with the original MID data, I use a version of MID data by Gibler, Miller & Little (2016) which revises the original data by dropping cases that do not meet the MID coding rules and making hundreds of either major or minor changes to the disputes. I use the peacesciencer package (version 1.0.0) by Miller (2022) to generate the MIDs data. I code a dyad year as experiencing costly conflict onset when there is a new dispute originated by both sides with hostility level above or equal to 4 (use of force). I lead the conflict variable by one year to alleviate potential concerns about spurious correlation. In the robustness check, I also rerun models using fatal MIDs (i.e. with at least one battlefield-related fatality).

### Bilateral strategic trade

My key independent variables are bilateral strategic trade and relative externality. I define strategic goods as commodities that are important for a country’s economic, technological, and military strength. This definition recognizes that aside from the economic benefits, a commodity is considered strategic only if it also holds technological and military implications.^11^ To code strategic commodities, I build on Goenner’s (2010) list of strategic goods which include energy, non-ferrous metals, chemicals, electronics, nuclear materials, and armaments. Under each of these categories, Goenner further identifies subcategories that are of strategic importance.

One potential limitation, however, is that some categories of manufactured goods are too broad and may not capture well particular commodities’ values to a country’s military and technology security. For instance, the list includes a broad category of telecommunications equipment and parts. But this category would include commodities such as electrical lines, microphones, and televisions that are not necessarily of much military and technology relevance. Therefore, I choose to further refine the categories of manufactured goods and only include goods that involve a high intensity of research and development (R&D).^12^ The refined list is shown in the Online appendix.

To code strategic trade, I use the Atlas of Economic Complexity’s version of Comtrade (The Growth Lab at Harvard University, 2019) which cleans the raw data’s inconsistent reports and covers states’ bilateral trade flows across different commodities from 1962 to 2017. Given my theory focuses on the beneficial aspects of dyadic trade (translating trade gains into military power), I choose to use dyadic strategic trade data (in constant 2010 US dollars) while controlling for each side’s yearly total trade.^13^ Given the skewness of the bilateral trade data, I log transform the trade variables.

### Relative externality

For the relative externality variable, further discussion over the security externality concept is in order. I define the security externalities of trade as states’ increased abilities to translate trade gains into (potential) military power due to the expansion of trade.^14^ While directly measuring the increased efficiency is challenging, we can proxy it by measuring the economic inputs and outputs that are relevant to states’ military power. Therefore, it is reasonable to measure trade externality via the aggregate volume of strategic goods. This also aligns with recent studies which suggest countries that consume and produce more strategic goods can more efficiently utilize trade gains (Fuhrmann, 2008; Goenner, 2010).

That said, there are two additional factors that are important to consider. First, simply tallying the amount of strategic goods is not sufficient to measure the externality concept because it will miss a state’s integration in the global trade networks. Economic integration is particularly important because losing access to foreign equipment and technology can increase the costs of or even impede military production and innovation (Paarlberg, 2004; Kennedy & Lim, 2018). India’s military modernization, for instance, relies heavily upon foreign weapon suppliers (Russia in particular). In 2006, aiming at improving its mobility the Indian army purchased 330 T-90 tanks from Russia with hundreds more for local assembly to enhance India’s indigenization of weapon production (Cohen & Dasgupta, 2010).

Economic integration also reflects states’ abilities to find alternative providers of strategic commodities and to endure economic containment (Kim, 2019). Relatedly, states’ integration levels can capture the underlying impact of economic sanctions or containment (that have been imposed). For instance, the trade embargoes by the West toward China and the heavy reliance on the Soviet Union during the 1950s and 1960s severely limited China’s ability to build up its industrial foundations (Liu, 2021). For another example, the Coordinating Committee for Multilateral Export Controls established by the Western Bloc during the Cold War proved to be quite tight and effective in limiting the Eastern Bloc’s access to advanced technology (Fergusson & Kerr, 2011). As the Cold Ward ended and the export control system switched to a much looser one (the Wassenaar Arrangement), many of these countries’ economic integration increased sharply. In this regard, states that are in a more central and integrated position in the global strategic trade networks are also more capable of translating trade gains into military power.

Second, as Dorussen (2006) pointed out, manufactured products have different security implications than non-manufactural goods. Building on this idea, it stands to reason that while imported strategic raw materials can be stored and used to improve a state’s security, exporting these goods could drain away the resources that states can tap into in times of conflict.^15^ East Asian countries such as Japan, China, and South Korea have been able to modernize their military because of their trade openness and the development of strong industrial bases. In comparison, Central Asian countries’ military modernization is more limited despite the rich natural resources the region holds (Tellis & Wills, 2005).

Meanwhile, although being able to import advanced technologies and strategic manufactured goods are important, it stands to argue that the abilities to export them can better reflect a state’s improvement in its defense and technology industrial base. The advancement of Chinese military modernization, for instance, is showcased by its reducing dependence on imports of sophisticated weapons and technologies and the increasing development and exports of indigenous technologies and products (Trebat & De Medeiros, 2014; cf. Gilli & Gilli, 2019). Therefore, in the main model I exclude the exports of strategic raw materials and imports of manufactured goods when calculating the externality measurement.^16^

Taking all three factors (strategic goods, economic integration, and manufactured goods) into account, I use the following formula to weigh the different commodities:


$$\text{E}xternality=\sum_{i}\text{S}trategic\;good{\text{s}}_{i}\times \text{I}ntegratio{\text{n}}_{i}$$

where *i* is a commodity identifier including two types: imports of strategic manufactured goods and exports of strategic manufactured goods. I identify the types of goods using the classification discussed above and log transform their yearly aggregates for each country. To proxy states’ integration, I use the trade centrality measurement accounting for the possibility of disconnected components (Zeng, 2020, 2021) and calculate for the above types of goods separately.^17^ According to this formula, states that trade in a higher volume and that are more integrated in the respective commodity networks will have a higher externality value. This way, I obtain the trade externality values for all country years.

**Figure 1.**
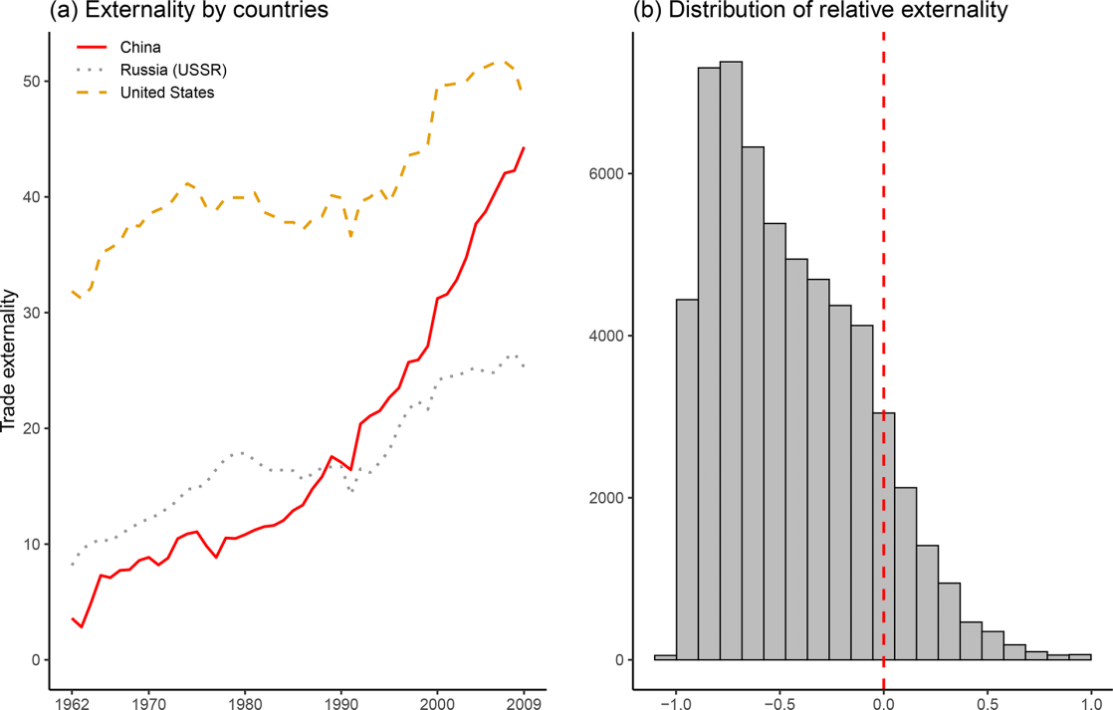
The externality measurement Panel (a) plots the externality values of three countries. Panel (b) plots the distribution of the relative externality measurement

As an example, I plot three countries’ externality values over time in Figure 1 (a) which provides some prima

facie evidence for the validity of the measurement.^18^ We can see that the USA is highly capable of exploiting the gains from trade. China, in comparison, starts from a low level but has witnessed an accelerated improvement, especially since the 1990s. As for Russia (USSR), its trade externality was higher than China’s before the mid-1980s but stagnated in the early 1990s. Finally, we also observe both Russia’s and the USA’s externalities experienced a discernible drop in 2009 while that was not the case for China.

With this trade externality measurement in hand, I subtract state 1’s externality by state 2’s and divide it by 2’s aggregates to proxy the relative externality variable. This way, the variable is scaled between –1 and 1. The distribution is shown in Figure 1 (b). As mentioned previously, state 1 is the relatively weaker side. When the relative externality measure is greater (smaller) than 0, it indicates the weaker side is more (less) capable of exploiting the gains from trade. We would therefore expect the distribution to be left-skewed (most weaker states are also less capable of exploiting the gains from trade) as is demonstrated by the histogram. Since the relative externality variable is aimed at capturing states’ expectations concerning potential power shifts, it stands to reason that a single year’s change may not necessarily lead to security concerns. Therefore, in the main models I use the five-year moving average to smooth out the trends.^19^

### Control variables

In addition to the two key independent variables, I add a number of control variables. I control for the minimum distance between two countries using the CShapes package (v0.6) in R (Weidmann, Kuse & Gleditsch, 2010) since geographic distance affects both trade and the

**Figure 2.**
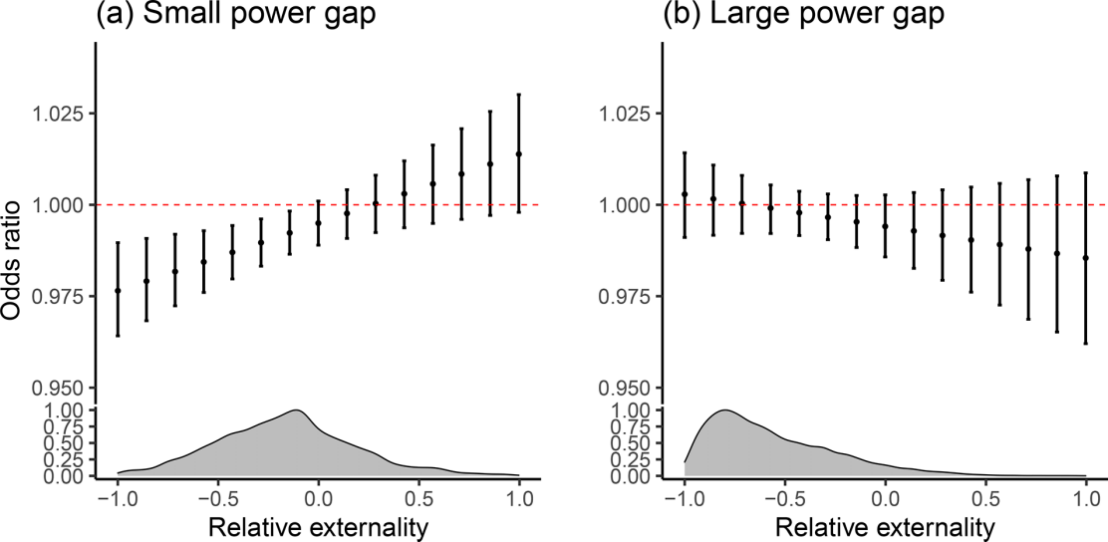
Marginal effects when increasing bilateral strategic trade by 10%, with 95% confidence intervals The x-axis denotes the value of relative externality, while the y-axis denotes the odds ratio. The distributions of relative externality are plotted for the respective samples with a small or large power gap.

effectiveness of military power; and the alliance status given allies tend to have more stable trade relations and that alliance can help alleviate concerns over commitment problems (Gibler, 2009).^20^ I control for whether both sides are democracies: the joint democracy variable is assigned a value of 1 when both sides have Polity scores equal to or greater than 6 (Marshall, Jaggers & Gurr, 2002). I account for the Cold War era (i.e. year before 1992). Finally, I also include the cubic polynomial of peace years as suggested by Carter & Signorino (2010).

Table I presents the summary statistics for variables used in the analysis. Consistent with existing knowledge, we see that states engaging in costly conflict tend to trade less, are closer to each other, and are less likely to be joint democracies. With these variables in hand, I specify logistic regression models interacting the bilateral strategic trade with the relative externality variables. I will run regressions separately for each sample. I will also include a model with three-way interaction which gives similar but more efficient estimates. In discussing the results, I will first focus on the regressions with two-way interaction given its ease of interpretation and then plot the marginal effects from the model with three-way interaction given its efficiency.

**Table I.** Summary statistics: 1962–2009

	*Costly conflict*		
*Variables*	*0, N = 47,834^a^*	*1, N = 534^a^*	*p-value^b^*
log Trade	3.29 (2.74)	3.09 (2.72)	0.023
Externality	–0.44 (0.35)	–0.26 (0.37)	<0.001
Trade 1	9.10 (2.01)	9.12 (1.95)	>0.9
Trade 2	12.24 (1.94)	10.69 (2.15)	<0.001
Distance	6.52 (3.28)	2.27 (3.32)	<0.001
Alliance			<0.001
0	37,859 (79%)	346 (65%)	
1	9,975 (21%)	188 (35%)	
Joint democracy			<0.001
0	34,354 (72%)	470 (88%)	
1	13,480 (28%)	64 (12%)	
Cold War			<0.001
0	24,381 (51%)	215 (40%)	
1	23,453 (49%)	319 (60%)	
Power gap			<0.001
Large	35,006 (73%)	199 (37%)	
Small	12,828 (27%)	335 (63%)	

^a^ Statistics presented: mean (SD); n (%).

^b^ Statistical tests performed: Wilcoxon rank-sum test; chi-square test of independence.

## Results

Table II presents the results of the logistic regressions. Model 1 is fitted for the sample with a small power gap; Model 2 is fitted for the sample with a large power gap. Model 3 is fitted for all politically relevant dyads

**Table II.** Logit regression with 95% confidence intervals: 1962–2009

	*Hostile MIDs*			
	*(1)*	*(2)*	*(3)*	*(4)*
log Trade	–0.029	–0.126*	–0.063	–0.088*
	(–0.099, 0.040)	(–0.240, –0.012)	(–0.156, 0.030)	(–0.170, –0.005)
log Trade × Rel externality	0.189**	–0.092	–0.094	
	(0.049, 0.328)	(–0.277, 0.094)	(–0.268, 0.080)	
log Trade × Power gap			0.010	
			(–0.084, 0.105)	
log Trade × Rel externality × Power gap			0.290*	
			(0.069, 0.510)	
Relative externality	–0.718†	–0.698	–0.316	–0.971†
	(–1.459, 0.023)	(–1.849, 0.453)	(–1.164, 0.533)	(–1.995, 0.054)
Power gap			0.392†	
			(–0.041, 0.825)	
Rel externality × Power gap			–0.589	
			(–1.420, 0.241)	
Trade 1	0.117†	0.360**	0.204**	0.364**
	(–0.021, 0.256)	(0.167, 0.554)	(0.093, 0.314)	(0.171, 0.557)
Trade 2	0.013	0.036	0.005	0.020
	( − 0.121, 0.148)	( − 0.151, 0.222)	( − 0.101, 0.112)	( − 0.163, 0.203)
Distance	− 0.223**	− 0.224**	− 0.223**	− 0.220**
	(–0.279, –0.167)	(–0.279, –0.170)	(–0.260, –0.185)	(–0.273, –0.166)
Alliance	–0.296*	0.116	–0.156	0.135
	(–0.552, –0.040)	(–0.256, 0.487)	(–0.366, 0.055)	(–0.236, 0.507)
Joint democracy	–0.344*	–0.606**	–0.469**	–0.657**
	(–0.651, –0.038)	(–1.006, –0.205)	(–0.711, –0.228)	(–1.048, –0.265)
Cold War	0.234†	0.301	0.240*	0.281
	(–0.024, 0.491)	(–0.062, 0.663)	(0.032, 0.448)	(–0.079, 0.641)
Peace year	–0.417**	–0.424**	–0.414**	–0.424**
	(–0.489, –0.345)	(–0.509, –0.339)	(–0.469, –0.360)	(–0.509, –0.339)
Peace year^2	0.019**	0.018**	0.018**	0.018**
	(0.014, 0.024)	(0.012, 0.023)	(0.014, 0.022)	(0.012, 0.023)
Peace year^3	–0.0003**	–0.0002**	–0.0002**	–0.0002**
	(–0.0004, –0.0002)	(–0.0003, –0.0001)	(–0.0003, –0.0002)	(–0.0003, –0.0001)
Constant	–2.406**	–5.141**	–3.457**	–5.117**
	(–3.354, –1.458)	(–6.770, –3.512)	(–4.357, –2.556)	(–6.747, –3.488)
Sample (power gap)	Small	Large	All	Large
BIC	2,398.88	1,937.452	4,275.951	1,928.02
Observations	12,521	32,268	44,789	32,268
Log Likelihood	–1,138.112	–901.244	–2,046.943	–901.719

†*p* < 0.1, *p < 0.05, **p < 0.01.

with three-way interaction among bilateral strategic trade, relative externality, and power gap. Finally, Model 4 is fitted for the sample with a large power gap but without interaction.

In Model 1, we see that the coefficient estimate for bilateral trade is negative (but insignificant) and the estimate for the interaction term between bilateral trade and relative externality is positive and significant. Given the range of the relative externality variable, this points to potential conflict-stoking effects of bilateral trade when the relative externality variable is large. Meanwhile, we can see that the coefficient estimate for bilateral trade is negative and significant and the estimate for the interaction term is negative but insignificant for Model 2. Taken together, these provide at least some tentative evidence for both hypotheses.

However, we do not know the direction, size, and uncertainty of the effects simply by reading off the results from the table. To more substantially demonstrate the impact, I plot the marginal effects of bilateral strategic trade from Model 3 while varying the value of relative externality from its lower to higher end in Figure 2.^21^ An odds ratio greater than 1 indicates the marginal effect is positive (i.e. the probabilities of conflict are increased). It is evident from the left panel that conditional effects hold when the power gap is small. This adds support to Hypothesis 1 for countries where the power imbalance is not extreme, increased bilateral strategic trade can promote peace when the relative externality is small. When the relative externality is large enough, the pacifying effects may dissipate.^22^

A cautious note concerning the support for Hypothesis 1, however, is in order. Specifically, we have a very small number of dyads with large relative externality values as showcased by the density plot; and the conflict-stoking effects at the far end in the left panel of Figure 2 are not significant. In this regard, it is more prudent to interpret the results as a lack of pacifying effects of bilateral strategic trade when the weaker side’s trade externality approaches or exceeds the stronger power’s.^23^

For the sample with a large power gap, I do not find evidence of conditional effects as shown in the right panel of Figure 2. To further examine whether the conditional effects hold, I run the likelihood ratio test between Model 2 and Model 4 and find no significant differences.^24^ Additionally, taking account of the lower Bayesian information criterion (BIC) value and the statistically significant effects of bilateral strategic trade in Model 4, it stands to reason for the sample with a large power gap the model without interaction provides a better fit of the data. Therefore, I find strong support for Hypothesis 2 for dyads with a large power gap – the effects of bilateral trade on costly conflict are negative and are not contingent on relative externality.

In addition to the above models, I perform robustness checks by: (a) adding yearly random effects; (b) adding yearly fixed effects; (c) using alternative externality measurement; (d) using peace year splines; (e) using fatal MIDs onset (i.e. MIDs with as least 1 fatality); (f) using rare event logistic regression; (g) using different moving averages (1, 3, 7, and 10-year) for the relative externality measure; (h) using different percentiles (top 35, 30, 20, and 15 percentiles) to separate the small vs. large power gap samples; (i) using fixed values of power ratio to separate the samples; (j) using an alternative measure of endogenous power; (k) running three-way interactions with continuous values of the power ratio variable; (l) adding additional size controls as suggested by Hegre (2009); (m) using the weaker side’s total strategic trade^25^ instead of bilateral strategic trade; (n) using an instrumental variable approach to account for potential endogeneity problems of bilateral trade as in Martin, Mayer & Thoenig (2008); (n) using the additive and multiplicative effects (AME) model as proposed by Minhas et al. (2021). The results are substantially similar to the main models’ and are shown in the Online appendix.

Synthesizing the above results, we can better understand why the pacifying effects of bilateral trade for China and the United States have been dissipating in recent years. The relative externality between China and the USA had been shrinking since 2008 and reversed sign (i.e. China as the relatively weaker power side became more efficient) in 2012. The underlying security concerns kick in and have been further exacerbated by the increasing military prowess of China. At the time of writing, the Biden administration has maintained Trump’s tariffs on imports from China and taken additional measures (e.g. the CHIPS and Science Act) aiming at countering China’s economic and security challenges. These measures, however, have been met by a defiant China taking more assertive approaches. For instance, in sharp contrast to its reticence toward Gingrich’s visit to Taiwan in 1997, China responded to Pelosi’s visit in 2022 by conducting unprecedented military exercises.

It is important to note that similar concerns – that the US leadership was threatened by other countries, Japan in particular, with ‘unfair trade’ practices – were also quite prominent in the 1980s. The Reagan administration adopted a wide range of protectionist tools to force these countries to deconstruct their industrial policies such as demanding a set of wide-ranging reforms within the Japanese semiconductor industry after 1985. For instance, the USA first demanded a 20% market share of US firms in the Japanese semiconductor market. And when seeing lack of progress, Reagan imposed a 100% tariff on $300 million worth of Japanese semiconductor exports to the USA (Wraight, 2019). Unlike the Chinese case, however, Japan acquiesced to most of the US demands.

Granted, there were many reasons behind Japan’s decision (e.g. their alliance relations and Japan’s reliance on US nuclear deterrence). My study suggests that one related reason was that Japan’s military power was not a threat to the USA in the first place. In this situation, Japan was willing to concede so as to retain the benefits of trade while the United States found it unnecessary to further escalate the threats or pressure. The above discussion suggests that increased bilateral strategic trade may no longer be peace-promoting if the externalities of trade aggravate concerns of adverse power shift. As mentioned at the beginning of this article, such security concerns are not limited to major powers. For instance, according to my measurement, Ukraine and Russia’s relative externality had been shrinking till 2014. Greece’s trade externality has been approximately equal to Turkey’s in recent decades. And although Myanmar is regarded as being militarily stronger than Thailand, the latter is much more efficient in exploiting trade gains. In all these examples, tensions have been simmering (or even exploded at times) despite the fact that they are major trade partners with each other.

## Conclusion

I have argued and shown that bilateral trade can empower countries, exacerbate security concerns, and ultimately increase the likelihood of costly conflict. The effects are contingent upon countries’ relative externality in translating trade gains into military power. Specifically, for countries where their existing military imbalance is not extreme, the pacifying effects of bilateral strategic trade can dissipate or even be reversed when the relatively weaker state becomes proportionally more capable of utilizing trade gains. By highlighting the link between trade, power, and security over time, I demonstrate the importance of investigating trade’s security externalities and expanding the current focus on opportunity costs.

That said, this article focuses on bilateral trade to re-examine the theoretical foundation. Future studies could explore how trade with third-party states further complicates the commitment problems (Peterson, 2011) and how alliance networks or security communities could further amplify a state’s externality (Beardsley et al., 2020). Additionally, although the possibility of destroying or capturing an opponent’s strategic commodities is not examined here, it is another important mechanism that may restrain trade’s pacifying effects. Finally, more empirical work aiming at refining the measures of states’ endogenous power can help us further investigate the indirect mediating mechanism: how trade or other economic factors shift states’ power over time which then affects the prospect of costly conflict.

## Notes

1 I focus on bilateral trade to investigate the theoretical foundation. See also footnote 25. See below on how this focus differs from previous concepts such as trade openness or trade dependence.

2 Based on the Direction of Trade Statistics (DOTS) by the IMF (https://data.imf.org/?sk=9D6028D4-F14A-464C-A2F2-59B2CD424B85, accessed 11 December 2021). The average annual growth rate in the 1980s was 17%, while the growth rate since 1990 is around 25.4%.

3 For instance, Germany fought against its major trade partners (Britain, France, Russia, and the USA) during World War I. India engaged in military conflict with its third major trade partner (China) in 2020. And Russia was among Georgia’s and Ukraine’s top trade partners before it fought wars against them in recent years.

4 I will focus on strategic trade throughout the rest of the article given its importance for a country’s technology and military strength. See the research design section for more details.

5 Other scope conditions include symmetry of dependence (Barbieri, 1996), commodity heterogeneity (Dorussen, 2006; Goenner, 2010), third-party trade (Crescenzi, 2003; Peterson, 2011), network effects (Kinne, 2012; Peterson, 2020), and membership in economic and security communities (Beardsley et al., 2020; Chen, 2020). Scholars also discuss the issue of reverse causality (Keshk, Pollins & Reuveny, 2004; Hegre, Oneal & Russett, 2010), the actual economic costs of conflict (Barbieri & Levy, 1999; Ward & Hoff, 2007; Long, 2008; Glick & Taylor, 2010), and whether multilateral trade openness increases the risks of conflict (Martin, Mayer & Thoenig, 2008; Yakovlev & Spleen, 2022).

6 Note that these studies focus on trade cooperation. See Powell (1991) and Morrow (1997) for critiques.

7 Studies of military buildups also indicate that asymmetric conflict could be operating on a very different logic (Sample, 2002). That said, the discussions here do not establish a priori how extreme power imbalance should be. In practice, we only have rough measurements of (potential) military power. In the empirical section, I present robustness checks on various operationalization of small vs. large power gaps.

8
Debs & Monteiro (2014) offer more detailed discussions concerning the need of using the endogenous components to examine power shifts. Using the original CINC index would bias toward larger countries too much. For instance, China would be identified as being stronger than the USA since the mid-1990s.

9 Using the 25 percentiles to separate the data would identify countries such as China, Russia, North Korea, and Vietnam as having a small power gap with the USA during certain years. If I use a higher benchmark, say the top 20 percentiles, it would only identify China and Russia. Lowering the benchmark to the top 30 percentiles would include Iraq in the 1980s and Turkey in the late 1990s.

10 For instance, North Korea would be ranked as the third most powerful state in 2016 by its large army and the lack of military spending data. In the robustness checks, I rerun the analysis with dichotomized power ratio variable by different percentiles or fixed values, three-way interaction models using the continuous power ratio variable, and an alternative measurement of endogenous power.

11 Some scholars argue that given resources are fungible, it may not be easy to identify strategic goods in practice. Baldwin, for instance, draws from Schelling (1958) and argues that any goods can be strategic so long as they are needed for consumption and that a state can not produce them in an efficient manner (Baldwin, 1985: 224). This is because they can save the state’s resources which then accrue to its economy. As such, Baldwin defines strategic goods as ‘goods and services for which (state) A has a relatively inelastic demand’ (Baldwin, 1985: 408). I choose not to define and measure strategic goods by trade elasticity because while it can capture a good’s economic importance, elasticity may not capture well the different values that commodities have on states’ technology and defense advancement. Salt, for instance, is an inelastic good. Most sneakers (that are not of luxury brands) are also inelastic in demand. But a state producing more salt or sneakers is not necessarily stronger in military or more advanced in technology.

12 I cross-check the commodities in Goenner’s list with the commodities identified in the Eurostat indicators on high-tech industry and knowledge-intensive services (https://ec.europa.eu/eurostat/cache/metadata/Annexes/htec_esms_an5.pdf, accessed 11 December 2021). It should be noted that Goenner’s list uses the Standard International Trade Classification (SITC) revision 2 code while the latter uses revision 4. Since revision 2 offers more coverage for earlier years, I convert the latter list’s codes back to revision 2. I also keep only the 4-digit code given the Atlas data’s coverage.

13 Many trade conflict studies use trade divided by GDP (or total trade) to capture a state’s economic dependence given their focus is on capturing the coercive aspects. In addition, weighing trade by each or both states’ trade volume or GDP could conflate the impact of bilateral trade with economic or country size (Mansfield & Pevehouse, 2000: 783).

14 This is similar to, though narrower than, Gowa & Mansfield’s (1993) definition of security externality as the increased economic efficiency due to trade, which frees more domestic resources for military uses. Using the broader definition would require a different operationalization of strategic goods (e.g. using trade elasticity). See footnote 11 for details.

15 Here and throughout the article, I use primary products and raw materials interchangeably even though some of the goods (non-ferrous metals) identified as raw materials are not necessarily primary products.

16 In the robustness checks, I also rerun the model including both types of trade. The results are substantially similar.

17 The Online appendix provides additional discussions concerning whether to weight the trade volumes by GDP or not in constructing the economic integration measure.

18 The Online appendix shows plots for more countries. I also provide further discussions concerning alternative operationalization choices. Specifically, instead of using log values of strategic trade to weight economic integration, I also tried using trade share, revealed comparative advantages as used in Kavakl, Chatagnier & Hatipoğlu (2020), normalized trade volumes over time, and strategic trade per capita.

19 In the robustness checks, I also rerun the analysis using 1, 3, 7, and 10-year moving averages.

20 I use the formal alliance data (v4.1) and code two countries as allies if they have signed a defense pact, neutrality, or non-aggression treaty. I exclude ententes given they only obligate members to consult and require little commitment.

21 The Online appendix provides plots using the results from Models 1 and 2 which showcase similar patterns as the plots from Model 3.

22 A stronger state may have interests in cutting off bilateral trade to reduce relative externality. Since it hurts both sides, it is not immediately clear that it would necessarily reduce relative externality. Even if it does, cutting off bilateral trade may not necessarily improve the prospect of peace. For the small power gap sample, we can imagine the marginal effects of cutting off bilateral strategic trade as the left panel of Figure 2 flipped along the red dashed line. In this regard, severing bilateral strategic trade before relative externality approach equity (value 0) can have positive marginal effects (i.e. increasing the risk of conflict).

23 That said, when I use a higher benchmark (top 15 percentiles and a power ratio value of 0.2) to separate the samples, the marginal effects of strategic bilateral trade for the sample with a small power gap can turn positive when relative externality is high.

24 By contrast, the likelihood ratio test between Model 1 and its nested model without interaction is statistically significant.

25 As one reviewer points out, the weaker side’s total trade could also be relevant to my theory. While I do not fully explore this given my focus, this robustness check indicates that could be a fruitful direction for future research.

## References

[bibr1-00223433231153902] Baldwin David Allen (1985) Economic Statecraft. Princeton, NJ: Princeton University Press.

[bibr2-00223433231153902] Barbieri Katherine (1996) Economic interdependence: A path to peace or a source of interstate conflict? Journal of Peace Research 33(1): 29–49.

[bibr3-00223433231153902] Barbieri Katherine Levy Jack S (1999) Sleeping with the enemy: The impact of war on trade. Journal of Peace Research 36(4): 463–479.

[bibr4-00223433231153902] Beardsley Kyle Liu Howard Mucha Peter J Siegel David A Tellez Juan F (2020) Hierarchy and the provision of order in international politics. Journal of Politics 82(2): 731–746.

[bibr5-00223433231153902] Blackwill Robert D Harris Jennifer M (2016) War by Other Means. Cambridge, MA: Harvard University Press.

[bibr6-00223433231153902] Bo Zhou (2020) The risk of China–US military conflict is worryingly high. Financial Times (https://www.ft.com/content/0f423616-d9f2-4ca6-8d3b-a04d467ed6f8, accessed 11 January 2021).

[bibr7-00223433231153902] Carter David B Signorino Curtis S (2010) Back to the future: Modeling time dependence in binary data. Political Analysis 18(3): 271–292.

[bibr8-00223433231153902] Chatagnier J Tyson Kavakl Kerim Can (2017) From economic competition to military combat: Export similarity and international conflict. Journal of Conflict Resolution 61(7): 1510–1536.

[bibr9-00223433231153902] Chen Frederick R (2020) Extended dependence: Trade, alliances, and peace. Journal of Politics 83(1): 246–259.

[bibr10-00223433231153902] Clarke Duncan L Johnston Robert J (1999) US dual-use exports to China, Chinese behavior, and the Israel factor: Effective controls? Asian Survey 39(2): 193–213.

[bibr11-00223433231153902] Cohen Stephen P Dasgupta Sunil (2010) Arming without Aiming: India’s Military Modernization. Washington, DC: Brookings Institution Press.

[bibr12-00223433231153902] Copeland Dale C (2015) Economic Interdependence and War. Princeton, NJ: Princeton University Press.

[bibr13-00223433231153902] Crescenzi Mark JC (2003) Economic exit, interdependence, and conflict. Journal of Politics 65(3): 809–832.

[bibr14-00223433231153902] Debs Alexandre Monteiro Nuno P (2014) Known unknowns: Power shifts, uncertainty, and war. International Organization 68(1): 1–31.

[bibr15-00223433231153902] Dorussen Han (2006) Heterogeneous trade interests and conflict: What you trade matters. Journal of Conflict Resolution 50(1): 87–107.

[bibr16-00223433231153902] *Economist* (2019) Trade can no longer anchor America’s relationship with China. The Economist (https://www.economist.com/special-report/2019/05/16/trade-can-no-longer-anchor-americas-relationship-with-china, accessed 11 November 2020).

[bibr17-00223433231153902] Fearon James D (2018) Cooperation, conflict, and the costs of anarchy. International Organization 72(3): 523–559.

[bibr18-00223433231153902] Fergusson Ian F Kerr Paul K (2011) The US Export Control System and the President’s Reform Initiative. Washington, DC: Library of Congress, Congressional Research Service.

[bibr19-00223433231153902] Fravel M Taylor (2019) Active Defense. Princeton, NJ: Princeton University Press.

[bibr20-00223433231153902] Fuhrmann Matthew (2008) Exporting mass destruction? The determinants of dual-use trade. Journal of Peace Research 45(5): 633–652.

[bibr21-00223433231153902] Gaddis John Lewis (2005) Strategies of Containment: A Critical Appraisal of American National Security Policy During the Cold War. Oxford: Oxford University Press.

[bibr22-00223433231153902] Gartzke Erik Li Quan Boehmer Charles (2001) Investing in the peace: Economic interdependence and international conflict. International Organization 55(2): 391–438.

[bibr23-00223433231153902] Gent Stephen E Crescenzi Mark JC (2021) Market Power Politics: War, Institutions, and Strategic Delay in World Politics. Oxford: Oxford University Press.

[bibr24-00223433231153902] Gibler Douglas M (2009) International Military Alliances, 1648–2008. Washington, DC: CQ.

[bibr25-00223433231153902] Gibler Douglas M Miller Steven V Little Erin K (2016) An analysis of the Militarized Interstate Dispute (MID) dataset, 1816–2001. International Studies Quarterly 60(4): 719–730.

[bibr26-00223433231153902] Gilli Andrea Gilli Mauro (2019) Why China has not caught up yet: Military-technological superiority and the limits of imitation, reverse engineering, and cyber espionage. International Security 43(3): 141–189.

[bibr27-00223433231153902] Gilpin Robert (1981) War and Change in World Politics. Cambridge: Cambridge University Press.

[bibr28-00223433231153902] Glick Reuven Taylor Alan M (2010) Collateral damage: Trade disruption and the economic impact of war. Review of Economics and Statistics 92(1): 102–127.

[bibr29-00223433231153902] Goenner Cullen F (2010) From toys to warships: Interdependence and the effects of disaggregated trade on militarized disputes. Journal of Peace Research 47(5): 547–559.

[bibr30-00223433231153902] Gowa Joanne (1994) Allies, Adversaries, and International Trade. Princeton, NJ: Princeton University Press.

[bibr31-00223433231153902] Gowa Joanne Mansfield Edward D (1993) Power politics and international trade. American Political Science Review 87(2): 408–420.

[bibr32-00223433231153902] Grieco Joseph M (1990) Cooperation among Nations: Europe, America, and Non-Tariff Barriers to Trade. Ithaca, NY: Cornell University Press.

[bibr33-00223433231153902] Hegre Håvard (2009) Trade dependence or size dependence? The gravity model of trade and the liberal peace. Conflict Management and Peace Science 26(1): 26–45.

[bibr34-00223433231153902] Hegre Håvard Oneal John R Russett Bruce (2010) Trade does promote peace: New simultaneous estimates of the reciprocal effects of trade and conflict. Journal of Peace Research 47(6): 763–774.

[bibr35-00223433231153902] Kavakl Kerim Can Tyson Chatagnier J Emre Hatipoğu (2020) The power to hurt and the effectiveness of international sanctions. Journal of Politics 82(3): 879–894.

[bibr36-00223433231153902] Kennedy Andrew B Lim Darren J (2018) The innovation imperative: Technology and US–China rivalry in the twenty-first century. International Affairs 94(3): 553–572.

[bibr37-00223433231153902] Keshk Omar MG Pollins Brian M Reuveny Rafael (2004) Trade still follows the flag: The primacy of politics in a simultaneous model of interdependence and armed conflict. Journal of Politics 66(4): 1155–1179.

[bibr38-00223433231153902] Kim Dong Jung (2019) Economic containment as a strategy of great power competition. International Affairs 95(6): 1423–1441.

[bibr39-00223433231153902] Kinne Brandon J (2012) Multilateral trade and militarized conflict: Centrality, openness, and asymmetry in the global trade network. Journal of Politics 74(1): 308–322.

[bibr40-00223433231153902] Krainin Colin (2017) Preventive war as a result of long-term shifts in power. Political Science Research and Methods 5(1): 103–121.

[bibr41-00223433231153902] Larson Deborah Welch Shevchenko Alexei (2010) Status seekers: Chinese and Russian responses to US primacy. International Security 34(4): 63–95.

[bibr42-00223433231153902] Lektzian David J Sprecher Christopher M (2007) Sanctions, signals, and militarized conflict. American Journal of Political Science 51(2): 415–431.

[bibr43-00223433231153902] Liu Lei (2021) China’s large-scale importation of western technology and the US response, 1972–1976. Diplomatic History 45: 794–820.

[bibr44-00223433231153902] Long Andrew G (2008) Bilateral trade in the shadow of armed conflict. International Studies Quarterly 52(1): 81–101.

[bibr45-00223433231153902] Mansfield Edward D Pevehouse Jon C (2000) Trade blocs, trade flows, and international conflict. International Organization 54(4): 775–808.

[bibr46-00223433231153902] Marshall Monty G Jaggers Keith Gurr Ted Robert (2002) Polity IV Project. College Park, MD: Center for International Development and Conflict Management at the University of Maryland College Park.

[bibr47-00223433231153902] Martin Philippe Mayer Thierry Thoenig Mathias (2008) Make trade not war? Review of Economic Studies 75(3): 865–900.

[bibr48-00223433231153902] Mearsheimer John J (1992) Disorder restored. In: Allison Graham Treverton Gregory F (eds) Rethinking America’s Security. New York: WW Norton, 213–237.

[bibr49-00223433231153902] Mearsheimer John J (2019) Bound to fail: The rise and fall of the liberal international order. International Security 43(4): 7–50.

[bibr50-00223433231153902] Meijer Hugo (2016) Trading with the Enemy: The Making of US Export Control Policy toward the People’s Republic of China. Oxford: Oxford University Press.

[bibr51-00223433231153902] Miller Steven V (2022) {peacesciencer}: An R package for quantitative peace science research. Conflict Management and Peace Science 39(6): 755–779.

[bibr52-00223433231153902] Minhas Shahryar Dorff Cassy Gallop Max B Foster Margaret Liu Howard Tellez Juan Ward Michael D (2021) Taking dyads seriously. Political Science Research and Methods 10(4): 703–721.

[bibr53-00223433231153902] Monteiro Nuno P Debs Alexandre (2020) An economic theory of war. Journal of Politics 82(1): 255–268.

[bibr54-00223433231153902] Morrow James D (1997) When do ‘relative gains’ impede trade? Journal of Conflict Resolution 41(1): 12–37.

[bibr55-00223433231153902] Organski Abramo FK Kugler Jacek (1980) The War Ledger. Chicago, IL: University of Chicago Press.

[bibr56-00223433231153902] Paarlberg Robert L (2004) Knowledge as power: Science, military dominance, and US security. International Security 29(1): 122–151.

[bibr57-00223433231153902] Peterson Timothy M (2011) Third-party trade, political similarity, and dyadic conflict. Journal of Peace Research 48(2): 185–200.

[bibr58-00223433231153902] Peterson Timothy M (2014) Dyadic trade, exit costs, and conflict. Journal of Conflict Resolution 58(4): 564–591.

[bibr59-00223433231153902] Peterson Timothy M (2020) Reconsidering economic leverage and vulnerability: Trade ties, sanction threats, and the success of economic coercion. Conflict Management and Peace Science 37(4): 409–429.

[bibr60-00223433231153902] Polachek Solomon Xiang Jun (2010) How opportunity costs decrease the probability of war in an incomplete information game. International Organization 64(1): 133–144.

[bibr61-00223433231153902] Powell Robert (1991) Absolute and relative gains in international relations theory. American Political Science Review 85(4): 1303–1320.

[bibr62-00223433231153902] Powell Robert (1999) In the Shadow of Power: States and Strategies in International Politics. Princeton, NJ: Princeton University Press.

[bibr63-00223433231153902] Sample Susan G (2002) The outcomes of military buildups: Minor states vs. major powers. Journal of Peace Research 39(6): 669–691.

[bibr64-00223433231153902] Schelling Thomas C (1958) International Economics. Boston, MA: Allyn & Bacon.

[bibr65-00223433231153902] Senese Paul D Vasquez John A (2008) The Steps to War: An Empirical Study. Princeton, NJ: Princeton University Press.

[bibr66-00223433231153902] Singer J David Bremer Stuart Stuckey John (1972) Capability distribution, uncertainty, and major power war, 1820–1965. In: Russett Bruce M (ed.) Peace, War, and Numbers. Beverly Hills, CA: Sage, 19–48.

[bibr67-00223433231153902] Tellis Ashley J Wills Michael (2005) Strategic Asia 2005-06: Military Modernization in an Era of Uncertainty. Seattle and Washington, DC: National Bureau of Asian Research.

[bibr68-00223433231153902] The Growth Lab at Harvard University (2019) The Atlas of Economic Complexity. Harvard University (http://www.atlas.cid.harvard.edu, accessed 11 November 2020).

[bibr69-00223433231153902] Trebat Nicholas M Medeiros Carlos Aguiar de (2014) Military modernization in Chinese technical progress and industrial innovation. Review of Political Economy 26(2): 303–324.

[bibr70-00223433231153902] Ward Michael D Hoff Peter D (2007) Persistent patterns of international commerce. Journal of Peace Research 44(2): 157–175.

[bibr71-00223433231153902] Weidmann Nils B Kuse Doreen Gleditsch Kristian Skrede (2010) The geography of the international system: The Cshapes dataset. International Interactions 36(1): 86–106.

[bibr72-00223433231153902] Weiss Jessica Chen (2022) The China trap: US foreign policy and the perilous logic of zero-sum competition. Foreign Affairs 101(5): 40–58.

[bibr73-00223433231153902] Wraight Tom (2019) From Reagan to Trump: The origins of US neoliberal protectionism. Political Quarterly 90(4): 735–742.

[bibr74-00223433231153902] Yakovlev Pavel Spleen Brandon (2022) Make concentrated trade not war? Review of Development Economics 26(2): 661–686.

[bibr75-00223433231153902] Zeng Yuleng (2020) Bluff to peace: How economic interdependence promotes peace despite increasing uncertainty and deception. Conflict Management and Peace Science 37(6): 633–654.

[bibr76-00223433231153902] Zeng Yuleng (2021) Biding time versus timely retreat: Asymmetric dependence, issue salience, and conflict duration. Journal of Peace Research 58(4): 719–733.

